# Interference with Oligomerization and Glycosaminoglycan Binding of the Chemokine CCL5 Improves Experimental Liver Injury

**DOI:** 10.1371/journal.pone.0036614

**Published:** 2012-05-04

**Authors:** Andreas Nellen, Daniel Heinrichs, Marie-Luise Berres, Hacer Sahin, Petra Schmitz, Amanda E. Proudfoot, Christian Trautwein, Hermann E. Wasmuth

**Affiliations:** 1 Medical Department III, University Hospital Aachen, Aachen, Germany; 2 Merck Serono, Geneva Research Centre, Geneva, Switzerland; Institute of Hepatology London, United Kingdom

## Abstract

**Background:**

The chemokine CCL5 is involved in the recruitment of immune cells and a subsequent activation of hepatic stellate cells (HSC) after liver injury. We here investigate whether inhibition of CCL5 oligomerization and glycosaminoglycan binding by a mutated CCL5 protein (^44^AANA^47^-CCL5) has the potential to ameliorate liver cell injury and fibrosis in vivo.

**Methodology:**

Liver injury was induced in C57BL/6 mice by intraperitoneal injection of carbon tetrachloride (CCl_4_) in an acute and a chronic liver injury model. Simultaneously, mice received either ^44^AANA^47^-CCL5 or vehicle. Liver cell necrosis and fibrosis was analyzed by histology, and measurement of serum transaminases and hydroxyproline. Intrahepatic mRNA expression of fibrosis and inflammation related genes were determined by quantitative RT-PCR and infiltration of immune cells was assessed by FACS analysis and immunocytochemistry. In vitro, HSC were stimulated with conditioned media of T-cell enriched splenocytes.

**Principal Findings:**

^44^AANA^47^-CCL5 treated mice displayed a significantly reduced degree of acute liver injury (liver cell necrosis, transaminases) and fibrosis (Sirus red positive area and hydroxyproline content) compared to vehicle treated mice. Ameliorated fibrosis by ^44^AANA^47^-CCL5 was associated with a decreased expression of fibrosis related genes, decreased α-smoth muscle antigen (αSMA) and a reduction of infiltrating immune cells. In the acute model, ^44^AANA^47^-CCL5 treated mice displayed a reduced immune cell infiltration and mRNA levels of TNF, IL-1 and CCL3 compared to vehicle treated mice. In vitro, conditioned medium of T-cell enriched splenocytes of ^44^AANA^47^-CCL5 treated mice inhibited the chemotaxis and proliferation of HSC.

**Conclusions:**

The results provide evidence that inhibition of oligomerization and glycosaminoglycan binding of the chemokine CCL5 is a new therapeutic strategy for the treatment of acute and chronic liver injuries and represents an alternative to chemokine receptor antagonism.

## Introduction

Acute and chronic liver diseases are a major cause of morbidity and mortality worldwide. In most diseases an inflammatory response within the liver is a mainstay of tissue damage [1]. After acute injury, an overwhelming immune response can lead to massive hepatocyte damage and subsequent liver failure [2]. On the other hand a continuous, low-level inflammation is a central pathophysiological aspect of liver fibrogenesis which ultimately leads to the development of liver cirrhosis in a significant number of cases [3]. Therefore, elucidation of pivotal inflammatory pathways in liver disease models might of great clinical interest as interference with these pathways bears the potential for new therapeutic options in diverse acute and chronic liver diseases.

The inflammatory infiltrate within the damaged liver consists of different immune cells subsets, including macrophages, dendritic cells, T cells, NK cells, NKT-cells and B-cells. Most of these cells are recruited into the liver along a chemotactic gradient. Classical chemoattractant molecules are chemokines, which are long known to govern the directed migration of leukocytes to sites of inflammation. In recent years, an important role of chemokines has also been deciphered in liver diseases [4]. Chemokines bind with high affinity to classical G-protein coupled receptors on the cell membrane of target cells and with lower affinity to glycosaminoglycans (GAG) of the extracellular matrix and endothelial cell surfaces [5]. Interaction of chemokines with GAG appears to be essential for the *in vivo* activity of certain chemokines and is considered as a prerequisite for establishing a chemotactic gradient across endothelial barriers [6]. Another biochemical characteristic of chemokines is their ability to form higher order oligomers what appears to be essential for their GAG binding and chemotactic activity *in vivo* [7].

CCL5 (traditional name: regulated upon activation, normally T cell expressed and secreted, RANTES) belongs to the CC family of inflammatory chemokines and is expressed by many liver resident and infiltrating cells [8]. In humans, CCL5 expression is up-regulated in acute and chronic liver damage [9], [10], [11] and functional *Ccl5* gene variants have been associated with inflammatory liver damage [12], [13] and treatment response [14]. Notably, in murine models of experimental fibrosis, CCL5 and its receptors CCR1 and CCR5 have been shown to be essential for fibrosis progression [9], [15]. Furthermore, antagonism of the CCL5 receptors with Met-CCL5 ameliorated liver fibrosis and accelerated the regression of scar formation in vivo [9]. Thus, this particular chemokine might be an attractive candidate for anti-inflammatory or anti-fibrotic therapies of liver diseases. However, chemokine receptor antagonism bears the potential for numerous unwanted side effects [16]. Therefore, other therapeutic strategies of chemokine antagonism should be systematically investigated.

Based on this background, we here investigate a novel therapeutic approach to interfere with the chemokine CCL5 in experimental liver damage models. We show that administration of the CCL5 mutant ^44^[AANA]^47^-CCL5, which loses the ability to oligomerise, but forms inactive heterodimers with wild-type CCL5 and loses 80% of its capacity to bind to GAG [17], strongly reduces acute liver injury and tissue fibrosis *in vivo*.

## Materials and Methods

### Murine *in vivo* experiments

Male wild-type (WT) mice on the C57BL/6 background (purchased from Charles River Laboratories) were subjected at the age of 8 weeks to two different liver damage models. In the first model, WT mice received a single intraperitoneal (i.p.) injection of carbon tetrachloride (CCl_4_, 0.8 mg/kg bodyweight) to induce acute liver injury. Animals were treated with the CCL5 antagonist [^44^AANA^47^]-CCL5 [17] or vehicle (PBS) i.p. before as well as 12 and 24 hours after liver damage. Mice were sacrificed 24 or 48 hours (n = 6/group) after the CCl_4_ administration for further analysis. In the second model, mice received repeated i.p. injections CCl_4_ for 6 weeks (0.6 mg/kg bodyweight in 1∶1 mineral oil, twice weekly) to induce liver fibrosis. Mice were sacrificed three days after the last CCl_4_ injection when the peek of fibrosis is expected [9]. [^44^AANA^47^]-CCL5 (10 µg daily) or vehicle (PBS, n = 12/group) were administered i.p. concomitantly to CCl_4_. In all mice, serum samples were obtained via cardiac puncture and livers were harvested for histological and biochemical analysis.

All animals were housed under specific-pathogen-free conditions in the animal facility of the RWTH University hospital Aachen. The experiments were carried out after approval by the animal welfare board at the Bezirksregierung Cologne, Germany (permit number 50.203.2-AC 5a, 51/06).

### Histological and biochemical evaluation of liver damage

Liver fibrosis was assessed histologically by quantification of the Sirius-red positive area on 10 low power (40×) fields/slide by use of the NIH software Image J, which is available from http://rsbweb.nih.gov/. Hepatic collagen content was analyzed biochemically by photometric measurement of the collagen specific amino acid hydroxyproline as recently described [9]. The intrahepatic expression of α-smooth muscle antigen (α-SMA) was assessed histologically with a mouse anti-SMA (Clone1A4, Dako) antibody. Immunohistochemical stainings for immune cell infiltration were performed in cooperation with the Institute of Pathology of University Hospital Bonn, Germany. The following antibodies were used: CD45, rat Clone30-F11 (BD Sciences); CD3, rabbit Clone SP7 (LabVision); F4/80, rat Clone BM8 (Dianova).

### Hepatic immune cell isolation and flow cytometry analysis

Single-cell suspensions were isolated from freshly harvested livers using mechanical and enzymatic digestion. Viable white blood cells were purified from the suspension by centrifugation for 20 min at 800× g with a density gradient separation medium (Lymphocyte, PAA Laboratories). PBMCs were collected from the gradient/supernatant interface and then washed in Hank's balanced salt solution (PAA Laboratories) supplemented with 1% bovine serum albumin and 2 mM ethylene diamine tetraacetic acid (EDTA). For flow cytometry analysis, cells were stained with fluorochrome-conjugated antibodies for CD3, CD4, CD8, CD45 and NK1.1 (all antibodies from Bioscience) and the relative numbers were quantified using the FACSCanto II (Becton Dickinson). Data were analyzed using FlowJo software (Tree Star).

### α-SMA Western blot

Snap-frozen liver samples were homogenised in 1 ml RIPA buffer (20 mM Tris-HCl, 150 mM NaCl, 2% Nonidet P40, 0.1% SDS, 0.5% Na-deoxycholate) with proteinase inhibitor (Mini Complete Protease Inhibitor Cocktail Tablets, Roche Applied Science) for isolation of total protein. Western blotting of α-SMA was performed from total liver protein, using a monoclonal mouse anti-mouse α-SMA antibody (Millipore). The primary antibody was visualized using horseradish peroxidase–conjugated anti-mouse IgG (DAKO) and the PIERCE ECL Western Blotting Substrate (Thermo Scientific). β-actin was used as loading control.

### mRNA expression analysis of murine fibrogenic and inflammatory genes

Total RNA was isolated from livers with peqGold TriFast (Peqlab) and reversely transcribed using RevertAid Premium FS cDNA Synthesis Kit (Fermentas) following the manufacturer's instructions. Quantitative RT-PCR was carried out for *Col1a1*, *Timp1, Tgf-β, IL-1β, TNF-α, Ccl3 and Ccl5* with Assays on Demand obtained from http://www.appliedbiosystems.com.

### Splenocyte isolation and preparation of conditioned media

Splenocytes from CCl_4_ alone or CCl_4_ plus [^44^AANA^47^]-CCL5 treated WT mice (see above) were isolated using standard protocols. Cells were cultured for 24 hours in RPMI-1640 medium (PAA Laboratories) and transferred to another tissue culture flask. The cell concentration was adjusted to 8×10^5^ cells per ml. For conditioned media, splenocyte suspensions were incubated for 48 hours with 10 µg/ml Concanavalin A (Sigma-Aldrich). The supernatant was centrifuged for 10 min at 900× g to remove cells and debris.

### Cell migration assay

A cell migration assay was performed using a modified Boyden Chamber. The stellate cell line GRX [18] [9] (2.5×10^5^ cells/ml) was placed in the upper chamber in RPMI-1640 medium with 10% fetal calf serum (FCS) and exposed to conditioned media of splenocytes isolated from CCl_4_ or CCl_4_ plus [^44^AANA^47^]-CCL5 treated WT mice in the lower chamber. After 4 hours of incubation at 37°C, cells that migrated to the lower chamber were counted in 6 randomly chosen (magnification, ×100) fields. All experiments were replicated at least twice in quadruplicates.

### Cell proliferation assay

GRX cells were starved for 16 hours in RPMI-1640 medium without FCS and stimulated for 24 hours with conditioned media from splenocytes of treated and untreated WT mice (see above). As controls RPMI-1640 medium with 10% FCS respectively without FCS was used. Proliferation of cells was measured by colorimetric immunoassay (Cell Proliferation Elisa, Roche Applied Science) following the manufacturer's instructions.

### Statistical analysis

Data are given as means ± SEM. Continuous variables were compared by two-sided *t*-tests with Welch's correction in case of unequal variances. *P*-values less than 0.05 were considered significant in all analyses. Statistical tests were performed by GraphPad Prism 5.

## Results

### [^44^AANA^47^]-CCL5 ameliorates acute liver injury through reduced immune cell infiltration

**Figure 1:**
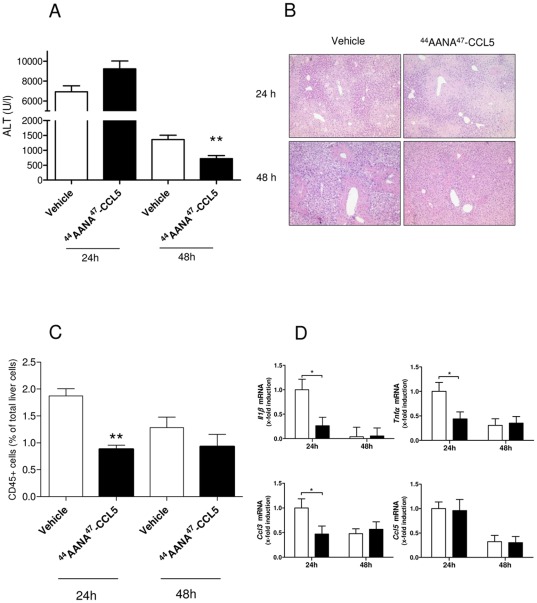
In vivo effects of treatment with [^44^AANA^47^]-CCL5 on acute liver cell injury. (**A**) Mice treated with [^44^AANA^47^]-CCL5 have reduced serum transaminases 48 hours after CCl_4_ application compared to vehicle treated mice. (**B**) Reduced liver cell damage is also evidenced by reduced necrotic areas 48 h hours after injury (HE staining, ×100). (**C**) The ameliorated damage after 48 h in [^44^AANA^47^]-CCL5 treated mice is preceded by a significantly reduced infiltration of CD45+ immune cells. (**D**) Reduced inflammatory liver damage in [^44^AANA^47^]-CCL5 is also associated with significantly repressed pro-inflammatory mRNA expression (black bars: [^44^AANA^47^]-CCL5, open bars: vehicle). * *P*<0.05, ** *P*<0.01.

We first subjected mice to a single toxic insult with CCl_4_ to induce acute liver injury. Mice were treated with either [^44^AANA^47^]-CCL5 or vehicle to assess the effects of interfering with CCL5 oligomerization and glycosaminoglycan binding on the severity of acute liver damage in this model. As depicted in figure 1A, administration [^44^AANA^47^]-CCL5 concomitantly to CCl_4_ resulted in reduced ALT levels 48 h after injury compared to vehicle treated mice (*P*<0.01). Interestingly, [^44^AANA^47^]-CCL5 did not modulate the ALT levels at 24 h what might mirror a direct cytotoxic effect of CCl_4_ which is not modifiable by immune interventions. Furthermore, it should be noted that the ALT levels 48 h after CCl_4_ administration are still much higher that the ALT levels in naïve animals. The same results were obtained for AST levels (data not shown). Decreased hepatocellular damage at 48 h was also reflected by a lower extend of hepatic necrosis as assessed by histology (Fig. 1B). Since [^44^AANA^47^]-CCL5 is thought to mainly inhibit the recruitment of leukocytes by reduced CCL5 binding to endothelial cells [19], we next analyzed the overall inflammatory infiltrate in the damaged livers. Indeed, treatment of the mice with [^44^AANA^47^]-CCL5 significantly reduced the overall inflammatory cell infiltration 24 h after CCl_4_ administration, as assessed by FACS counting of CD45+ cells, compared to vehicle treated animals (Fig. 1C, *P*<0.01). Reduced inflammatory liver damage was further evidenced by RT-PCRs for pivotal inflammation-related genes in mice with or without [^44^AANA^47^]-CCL5 treatment (Fig. 1D). Specifically, a significantly reduced hepatic mRNA expression of *IL-1β*, *Tnf-α* and the chemokine *Ccl3* preceded the ameliorated ALT levels in [^44^AANA^47^]-CCL5 compared to vehicle treated mice. Interestingly, the mRNA expression of CCL5 was not different between the two groups, supporting that compensatory CCL5 synthesis is not induced by [^44^AANA^47^]-CCL5.

### Liver fibrosis is strongly reduced in [^44^AANA^47^]-CCL5 treated animals

**Figure 2:**
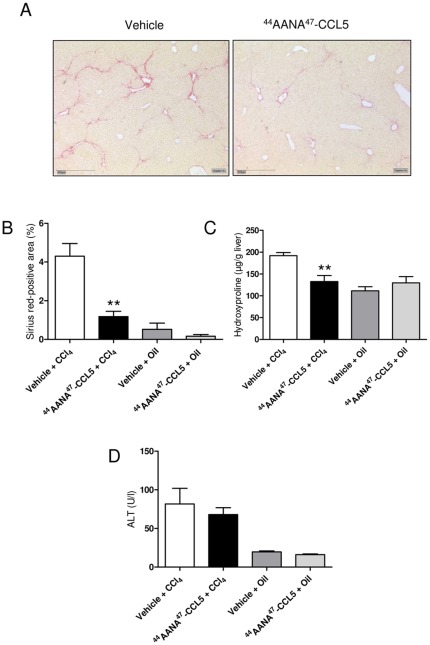
In vivo effects of treatment with [^44^AANA^47^]-CCL5 on chronic liver injury. (**A**) Treatment of mice with [^44^AANA^47^]-CCL5 in parallel to chronic CCl_4_ intoxication reduced scar formation in the liver compared to vehicle (representative Sirius red stainings, ×100). Ameliorated liver fibrosis in [^44^AANA^47^]-CCL5 treated mice is quantified by a reduced Sirius red positive area (**B**) and a decreased hepatic concentration of the collagen specific amino acid hydroxyproline (**C**). ALT levels were not significantly changed in [^44^AANA^47^]-CCL5 treated mice compared to vehicle treated mice when taken 72 h after the last CCl_4_ injection (**D**). ** *P*<0.01.

**Figure 3:**
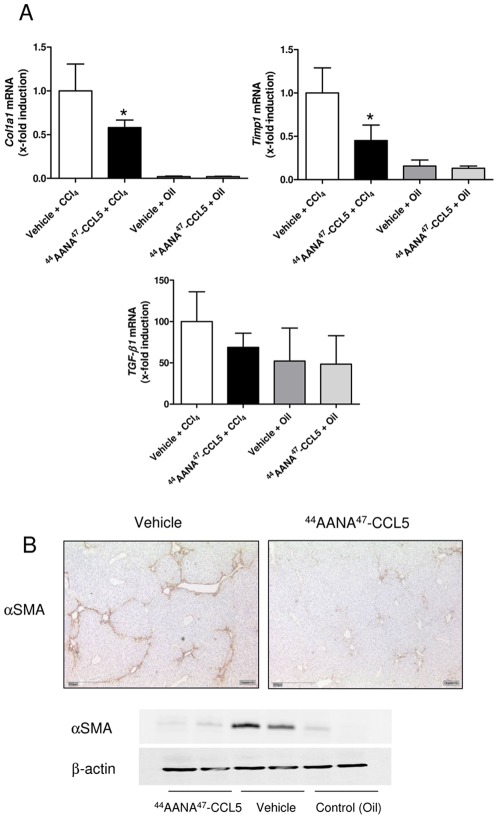
Treatment with [^44^AANA^47^]-CCL5 is associated with reduced stellate cell activation. (**A**) Mice treated with [^44^AANA^47^]-CCL5 concomitantly to CCl4 display repressed mRNA expressions of the pivotal stellate cell genes *Col1a1* and *Timp1* compared to vehicle treated animals, while the mRNA of Tgf-β showed only a trend to lower expression. (**B**) Reduced activation of stellate cells in [^44^AANA^47^]-CCL5 treated mice is further evidenced by decreased protein expression of the stellate cell activation marker αSMA by immunohistochemistry (upper panels) and western bot of total liver extracts (lower panel). * *P*<0.05.

After having shown that treatment with [^44^AANA^47^]-CCL5 has a strong impact on the extent of acute inflammatory liver damage, we next evaluated whether this antagonistic strategy does also inhibit progression of liver fibrosis induced by repetitive CCl_4_ intoxications. As depicted in figure 2A, mice treated with [^44^AANA^47^]-CCL5 indeed showed a reduced extend of scar formation compared to vehicle treated animals. The improved severity of fibrosis in the [^44^AANA^47^]-CCL5 treated animals was quantified by a significant decrease in the Sirius red-positive area (*P*<0.01, Fig. 2B) and a reduced hepatic concentration of the collagen-specific amino acid hydroxyproline (reduction of 30.1%, *P*<0.01, Fig. 2C), respectively. Although there was also a trend towards lower ALT levels, Furthermore, the mRNA expression of the fibrosis-associated genes *Col1a1* and *Timp1* was significantly reduced in the livers of [^44^AANA^47^]-CCL5 compared to vehicle treated mice (both *P*<0.05, Fig. 3A). In addition, the mRNA expression of the important fibrogenic cytokine *Tgf-β* was reduced by 32% in [^44^AANA^47^]-CCL5 treated mice compared to vehicle treated mice, although this difference did not reach statistical significane due to the high variation of expression within the groups. Since *Timp1* is mainly expressed by hepatic stellate cells, we also assessed the activation status of these pivotal pro-fibrogenic cells [20] by determination of αSMA expression. As shown in figure 3B, treatment of mice with [^44^AANA^47^]-CCL5 resulted in a reduced expression of αSMA as assessed by immunohistochemistry and western blot analysis of total αSMA protein in the liver. Thus, the reduced fibrosis phenotype in [^44^AANA^47^]-CCL5 treated mice appeared to be associated with a strongly reduced activation of hepatic stellate cells.

### [^44^AANA^47^]-CCL5 leads to quantitative and qualitative changes in the immune response following CCl_4_ administration

**Figure 4:**
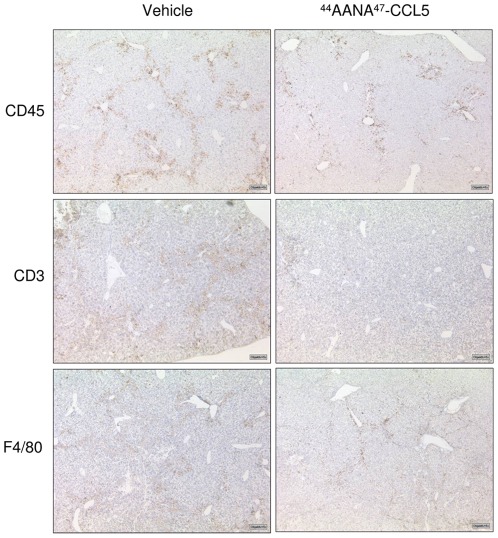
Treatment with [^44^AANA^47^]-CCL5 leads to altered immune cell infiltration during liver fibrogenesis. The photomicrographs show representative liver sections of mice treated with [^44^AANA^47^]-CCL5 or vehicle after staining for CD45 (total leukocytes), CD3 (T-cells) and macrophages (F4/80). Magnification ×100.

We and others have recently shown that hepatic stellate cells are a main target of CC chemokines during liver fibrogenesis which are mainly secreted by liver infiltrating immune cells [9], [15]. Thus, we next analysed whether treatment with [^44^AANA^47^]-CCL5 also leads to reduced immune cell infiltration during chronic liver injury with CCl_4_. Indeed, the infiltration of CD45+ leukocytes was strongly reduced in [^44^AANA^47^]-CCL5 compared to vehicle treated mice (Fig. 4, upper panel). Since chemokines, including CCL5, are mainly secreted by T-cells and monocytes/macrophages during liver fibrogenesis [21], we specifically determined the recruitment of these cells into the liver at peak of fibrosis. As depicted in figure 4, the infiltration of both cell populations was indeed reduced in [^44^AANA^47^]-CCL5 treated mice as assessed by immunohistochemistry for CD3 and F4/80, respectively. As CCL5 is a main chemoattractant for both of these cell populations [22], these results are well in line with the proposed action of [^44^AANA^47^]-CCL5 which has abrogated glycosaminoglycan binding and forms inactive heterodimers with WT-CCL5 thereby inhibits transendothelial migration of immune cells induced by CCL5.

**Figure 5:**
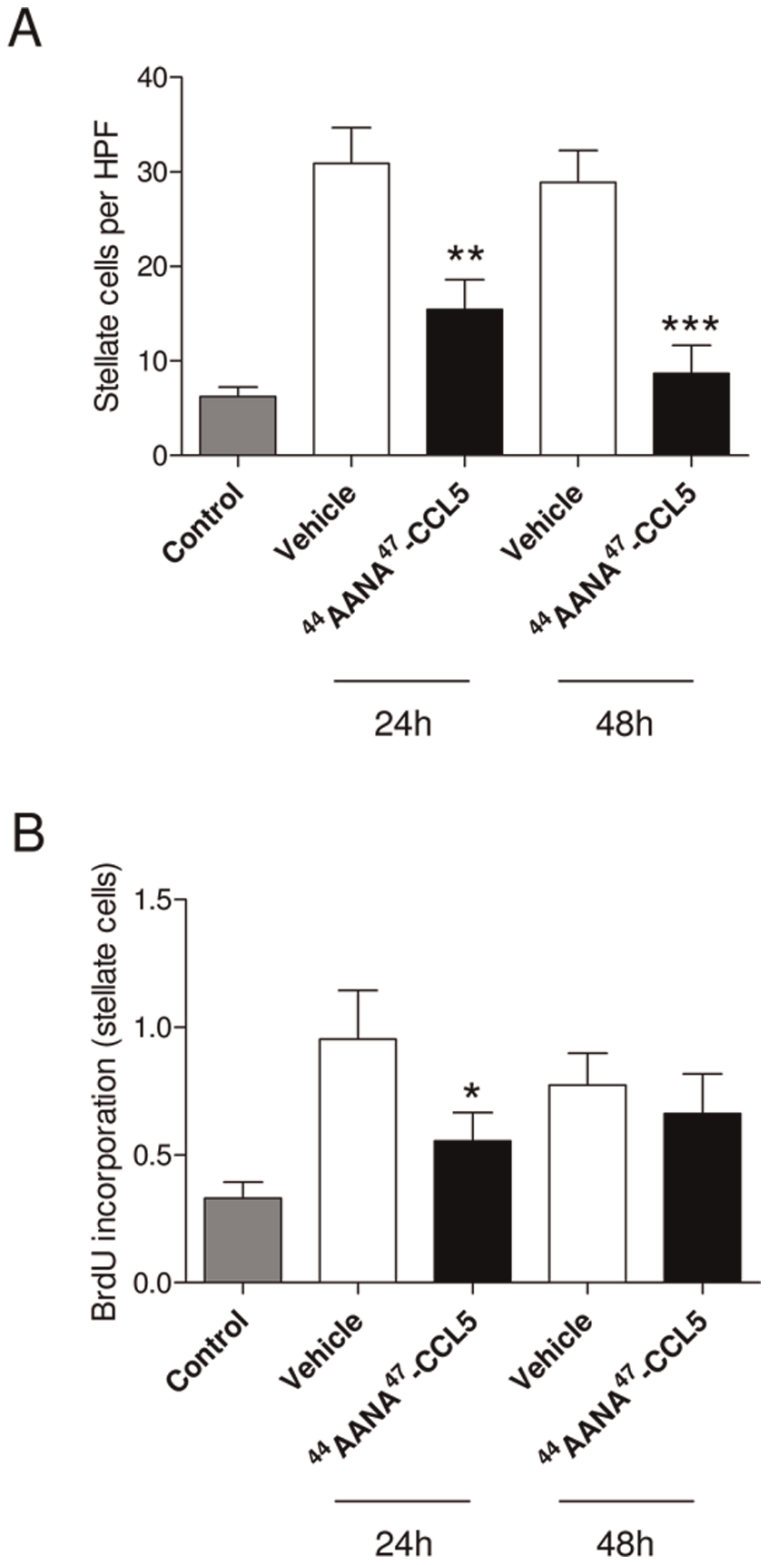
Treatment with [^44^AANA^47^]-CCL5 leads to qualitative changes in splenocytes. (**A**) Splenocytes were isolated from [^44^AANA^47^]-CCL5 and vehicle treated mice and conditioned mediate of splenocyte cultures were assessed for stellate cells migration in a Boyden chamber. Conditioned medium of splenocytes isolated from [^44^AANA^47^]-CCL5 treated mice (24 and 48 h after CCl_4_ application) was significantly less effective in inducing stellate cell migration compared to splenocytes isolated from vehicle treated mice. (**B**) Conditioned medium from mice treated [^44^AANA^47^]-CCL5 was also less effective in inducing stellate cell proliferation as assessed by BrdU incorporation. * *P*<0.05, ** *P*<0.01.

Apart from these quantitative changes in immune cell subsets, we also assessed whether [^44^AANA^47^]-CCL5 has an impact on qualitative aspects of the immune response. To this end, splenocytes of [^44^AANA^47^]-CCL5 or vehicle treated mice were isolated and the direct effects of conditioned media of these cells after ConA stimulation on hepatic stellate cells were analyzed. The results are presented in figure 5. Compared to vehicle treated mice, conditioned medium from [^44^AANA^47^]-CCL5 treated mice significantly reduced the migration (Fig. 5A) and the proliferation (Fig. 5B) of hepatic stellate cells *in vitro*. As both aspects are important parameters of these cells during fibrogenesis [20], [^44^AANA^47^]-CCL5 appears to also impact the biology of these cells by modulating qualitative changes of the immune response outside the liver.

## Discussion

The findings presented in the current study further establish an important role of the CC chemokine CCL5 in acute and chronic liver diseases. There is now ample evidence that this particular chemokine and its receptors play a functional role in murine models of acute [23] and chronic liver diseases [9], [15]. Furthermore, expression data and genetic analysis also suggest that CCL5 is involved in the pathogenesis of liver diseases in humans [12], [13], [14], [24]. Thus, CCL5 might be an attractive candidate for antagonistic studies to inhibit the progression and/or accelerate the regression of chronic liver diseases. Indeed, we could recently show that administration of an amino-terminally modified CCL5 molecule (Met-CCL5 [25]), which is an antagonist of the CCL5 receptors CCR1 and CCR5, is able to ameliorate experimental liver damage in mice [9]. However, this particular antagonist blocks not only the action of CCL5 but also of other ligands of these chemokine receptors, including CCL3 and CCL4, which might have differential effects within and outside the liver [16]. We therefore investigated another principle of interference with the chemokine system in the current study which is not based on receptor antagonism, and might exhibit another pattern of side effects. The mutated chemokine [^44^AANA^47^]-CCL5 is known to interfere with heparin binding and oligomerization of CCL5, which are both important biological aspects of it's activity *in vivo* [26], [27]. This antagonist has been shown to limit inflammatory cell recruitment into the peritoneal cavity, bronchoalveolar air space and the CNS in mice, thereby ameliorating the phenotype of inflammation in these organs [17]. Furthermore, [^44^AANA^47^]-CCL5 is able to ameliorate myocardial reperfusion injury in atherosclerotic mice [19]. We here extend these earlier findings by showing that systemic administration of [^44^AANA^47^]-CCL5 is also able to strongly inhibit the infiltration of immune cells into the damaged liver. This therapeutical effect was true for an acute model of liver injury as well as a chronic model leading to liver fibrosis. As it is now well established that infiltrating immune cells play a pivotal role in both phenotypes of liver disease [28], these effects of [^44^AANA^47^]-CCL5 might be considered as the driving force behind the observed reduction of liver damage. Since T-cells and macrophages are known to carry receptors for CCL5 [29], we systematically addressed the infiltration of these cells into the liver by immunocytochemistry. As depicted in figure 4, the infiltration of both of these cell populations was indeed reduced in [^44^AANA^47^]-CCL5 compared to vehicle treated mice. As T-cells [30] and monocytes/macrophages [31] are considered as pivotal pro-fibrogenic cell populations, these results might largely explain the phenotype observed in our models. In support of this hypothesis immune cell populations which do not carry CCL5 receptors (e.g. B-cells) were not changed by treatment with [^44^AANA^47^]-CCL5 (data not shown). In the acute model of liver injury the reduced infiltration of immune cells was associated with a strong reduction of pivotal inflammatory genes, while in the chronic model reduced cell recruitment was linked to diminished HSC activation. This finding further emphasizes the important interplay between immune cells and HSCs during different types of liver injury [32].

Nevertheless, we also observed qualitative differences of immune mediated HSC activation in mice treated with [^44^AANA^47^]-CCL5. To this end, we isolated splenocytes from mice treated with [^44^AANA^47^]-CCL5 or vehicle and used the supernatant of these cultures for assessing the migration and proliferation of HSCs *in vitro*. Indeed, splenocyte supernatants obtained from mice treated with [^44^AANA^47^]-CCL5 were significantly less potent in inducing migration and proliferation of HSCs. Thus, the effects of [^44^AANA^47^]-CCL5 in our liver disease models appears to be mediated not only by quantitative effects of immune cell infiltration into the liver, but also by qualitative changes outside the liver. Whether this is due to the function of [^44^AANA^47^]-CCL5 to sequester endogenous CCL5 by forming non-functional heterodimers [17] needs to be analyzed in further studies. Furthermore, other cytokines might be involved in the modulation of stellate cell behaviour. In a first attempt to identify these cytokines, we determined a subset of these molecules in the conditioned media of activated splenocytes, but could not detect major differences between cells isolated [^44^AANA^47^]-CCL5 and vehicle treated mice (data not shown). Thus, the identification of the molecules involved in the modulation of HSC behaviour in our models is still warranted but beyond the scope of the current study.

In summary, the current results suggest that interference with oligomerization and glycosaminoglycan binding of the chemokine CCL5 is able to ameliorate experimental liver injury and fibrosis *in vivo*. Although we cannot decide from our data that this approach is associated with fewer side effects than direct chemokine receptor antagonism, this strategy of immune intervention might be further evaluated as a novel option for the treatment of liver diseases.
